# Control and Transfer of Chirality Within Well-Defined Tripodal Supramolecular Cages

**DOI:** 10.3389/fchem.2020.599893

**Published:** 2020-11-03

**Authors:** Gege Qiu, Paola Nava, Cédric Colomban, Alexandre Martinez

**Affiliations:** Aix Marseille Univ, CNRS, Centrale Marseille, iSm2, Marseille, France

**Keywords:** cages, chirality, transfer, tripodal, supramolecular

## Abstract

The development of new strategies to turn achiral artificial hosts into highly desirable chiral receptors is a crucial challenge in order to advance the fields of asymmetric transformations and enantioselective sensing. Over the past few years, *C*_3_ symmetrical cages have emerged as interesting class of supramolecular hosts that have been reported as efficient scaffolds for chirality dynamics (such as generation, control, and transfer). On this basis, this mini review, which summarizes the existing examples of chirality control and propagation in tripodal supramolecular cages, aims at discussing the benefits and perspectives of this approach.

## Introduction

Stereoselective bindings are essential events for biochemical functions such as enzymatic catalysis and recognition of natural metabolites. In order to reproduce the efficiency of the stereoselective receptors found in the biological world, the synthesis of chiral artificial hosts has attracted considerable attentions over the past few decades. In this context, the supramolecular approach appears as particularly promising as it allows for the construction of tridimensional hosts that could, for example, mimic enzyme cavities (Liu et al., 2015). Among supramolecular hosts, organic or self-assembled cages built from chiral building blocks are of particular interest due to their ability to bind guests within their interior. A variety of chiral organic (Brotin et al., 2013), or metallo-cages (Hardie, 2016; Chen et al., 2017) have been constructed over the past years, via covalent bond or coordination driven assembly processes, respectively. Such supramolecular cages have found a wide range of applications from selective recognition and separation of hydrocarbon derivatives (Zhang et al., 2018, 2019), chiral molecules (Brotin and Dutasta, 2009) and noble gases (Mastalerz, 2018), to drug delivery (Sepehrpour et al., 2019; Samanta and Isaacs, 2020), photophysical and CPL properties (Saha et al., 2016; Feng et al., 2018; Jing et al., 2019; Zhou et al., 2019), functional molecular machines (Oldknow et al., 2018; Elemans and Nolte, 2019), stabilization of reactive species (Mal et al., 2009) and catalysis in confined spaces (Hong et al., 2018; Mouarrawis et al., 2018; Roland et al., 2018).

However, being able to induce chirality dynamics (generation and propagation) within supramolecular cages and their related host-guest complexes remains a highly challenging task. In this line, *C*_3_ symmetrical cages present a structural advantage due to their propensity to form triple-stranded helix or propeller-like structures (Yamakado et al., 2013; Míguez-Lago et al., 2015; Malik et al., 2018; Sato et al., 2018). Aiming at providing a general view about recent progress in the preparation of chiral tripodal cages displaying chirality dynamics, this mini-review summarizes current knowledge on how the chiral information can propagate along such tridimensional architectures. Control of the chirality within tripodal cages upon chiral sorting (Henkelis et al., 2014; Schaly et al., 2016; Jedrzejewska and Szumna, 2017; Schulte et al., 2019; Séjourné et al., 2020), or guest binding (You et al., 2012; Bravin et al., 2019; Pavlovic et al., 2019) are beyond the scope of this work.

The first part of the mini-review will be devoted to examples of propagation of the stereochemical information from one chiral unit to linkers of *C*_3_ symmetrical cages. Its second part will describe how the chiral unit can control and induce a chiral arrangement of another tripodal unit included in the cage structure.

## Control of the Chiral Arrangement of the Linkers in Tripodal Cages

A straightforward strategy to generate chiral hosts consists in the covalent substitution of a chiral precursor, to create an inner cavity. In particular, chiral *C*_3_ symmetrical cages have been obtained by connecting one chiral moiety with another tripodal unit, by three linkers. The presence of three identical linkers connected to one chiral component can interestingly lead to the formation of triple-stranded helical structures with a controlled orientation. It has been indeed observed that the stereochemical information of the chiral unit could propagate along the structure to (i) induce a controlled propeller-like arrangement of the linkers or (ii) turn the other opposite moiety into a chiral structure with a controlled orientation.

**Figure 1:**
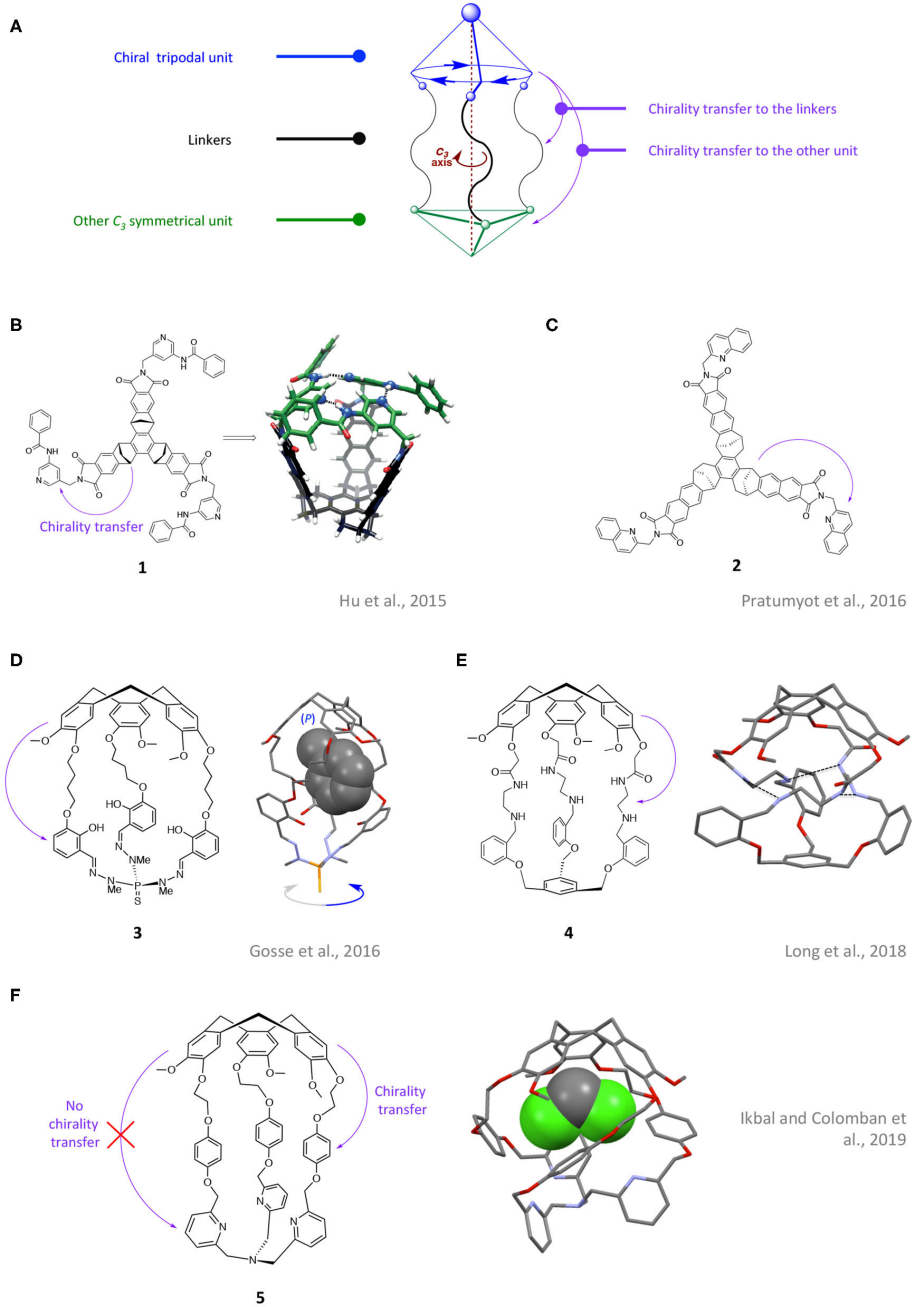
**(A)** Schematic representation of the structural characteristics of the chiral cages depicted in this review. Examples of tripodal cages displaying a transfer of chirality between a chiral unit and its side arms: schematic representation and computed structures of open cages **1 (B)** and **2 (C)** together with the schematic representation and XRD structures of hemicryptophanes **3 (D)**, **4 (E)** and **5 (F)** (the XRD structure of **3** and **5** displays an entrapped molecule of toluene and CH_2_Cl_2_ respectively). Purple arrows depict the observed chirality transfers.

Chirality transfer events within tripodal hosts were firstly evidenced between the chiral unit and its nearest linkers, resulting in a remote control of their helical arrangement (Figure 1). For example, Badjic et al. reported the preparation and characterization of the gated stereoisomeric basket (**1**) (Hu et al., 2015). Such *C*_3_ symmetrical cavitand was built from a basket unit owning a *P*- or *M*- twisted structure, decorated by three aminopyridine gates at its rim. The authors demonstrated, through a combination of ^1^H/^13^C-NMR analysis and computational results, that the aminopyridine substituents display a right- or left-handed propeller like arrangement maintained by an intramolecular N-H^…^N hydrogen-bond network. Interestingly, the unidirectional orientation of the gate folding is dictated by the chirality (*P*- or *M*-) of the southern twisted chiral basket. Computational studies further suggest that the *P*- basket framework imposes an anti-clockwise orientation while the *M*- basket results in a clockwise arrangement. By replacing aminopyridines by quinolone gates, the same team reported, 1 year later, the enantiopure basket (**2**), which displays a solvent dependent transfer of the stereochemical information from the basket to the rim (Pratumyot et al., 2016). By comparison with **1**, **2** exhibits π-stacked gates instead of hydrogen-bonded ones. 2D ^1^H-NMR characterizations, exciton-coupled circular dichroism (ECD) analysis, and computational modeling, reveal that the clockwise and/or anticlockwise orientations of the quinolone gates exist in acetonitrile while the three substituents remain randomly oriented in the non-polar dichloromethane solvent.

In the same vein, hemicryptophanes are organic cages built from a northern bowl-shaped, *C*_3_ symmetrical cyclotriveratrylene (CTV) unit, connected to another tripodal moiety by three spacers (Zhang et al., 2017). Due to the inherent chirality of the CTV unit, hemicryptophanes are chiral cages with *M* or *P* configuration. Enantiopure versions of this kind of host are commonly obtained following two main strategies: (i) the chiral HPLC resolution of a racemic mixture of *P* and *M* structures and (ii) the addition of another chiral moiety and separation of the resulting diastereoisomers (Colomban et al., 2019). Hemicryptophane (**3**), which connects a CTV unit and a phosphotrihydrazone moiety through three butylene (-C_4_H_8_-) linkers, was reported in 2016 as ligand for Ga^III^ and Fe^III^ metal ions (Gosse et al., 2016). The authors observed, through XRD analysis, that the butylene linkers of **3** displayed a clockwise/anticlockwise helical orientation (α or β helicity). Interestingly, for both **3** and its corresponding Ga^III^ and Fe^III^ complexes, such solid-state helical arrangement of the linkers (α or β) was imposed by the configuration of the CTV unit. Indeed only (*M*-)CTV-(β-)helix and (*P*-)CTV-(α-) helix pairs of enantiomers were observed. In 2018, the group of Martinez reported a new example of hemicryptophane cages displaying a remote control of the linkers' helical arrangement, dictated by the CTV unit (Long et al., 2018a). The hemicryptophane **4**, which displays linkers constituted of both amine and amide groups, was prepared. Analysis of its X-ray molecular structure reveals H-bond interactions between the amide and the amine function of each arm resulting in a triple-stranded helical arrangement of the linkers (Figure 1D). Interestingly, the chirality of such triple helices was dictated by the chirality of the CTV unit (*P*- or *M*-). Hemicryptophane **(*P*)-4** indeed displayed a Δ propeller-like arrangement of the linkers while **(*M*)-4** revealed a Λ orientation. Moreover, careful ^1^H-NMR analyses allow the authors to suggest that the controlled arrangement observed in the solid state may be retained in solution. This example highlights the remarkable flexibility of the whole organic structure that is strongly twisted and displays a propagation of the CTV chirality over nine bonds.

## Control of the Chiral Arrangement of an Other *C_3_* Unit in Tripodal Cages

**Figure 2:**
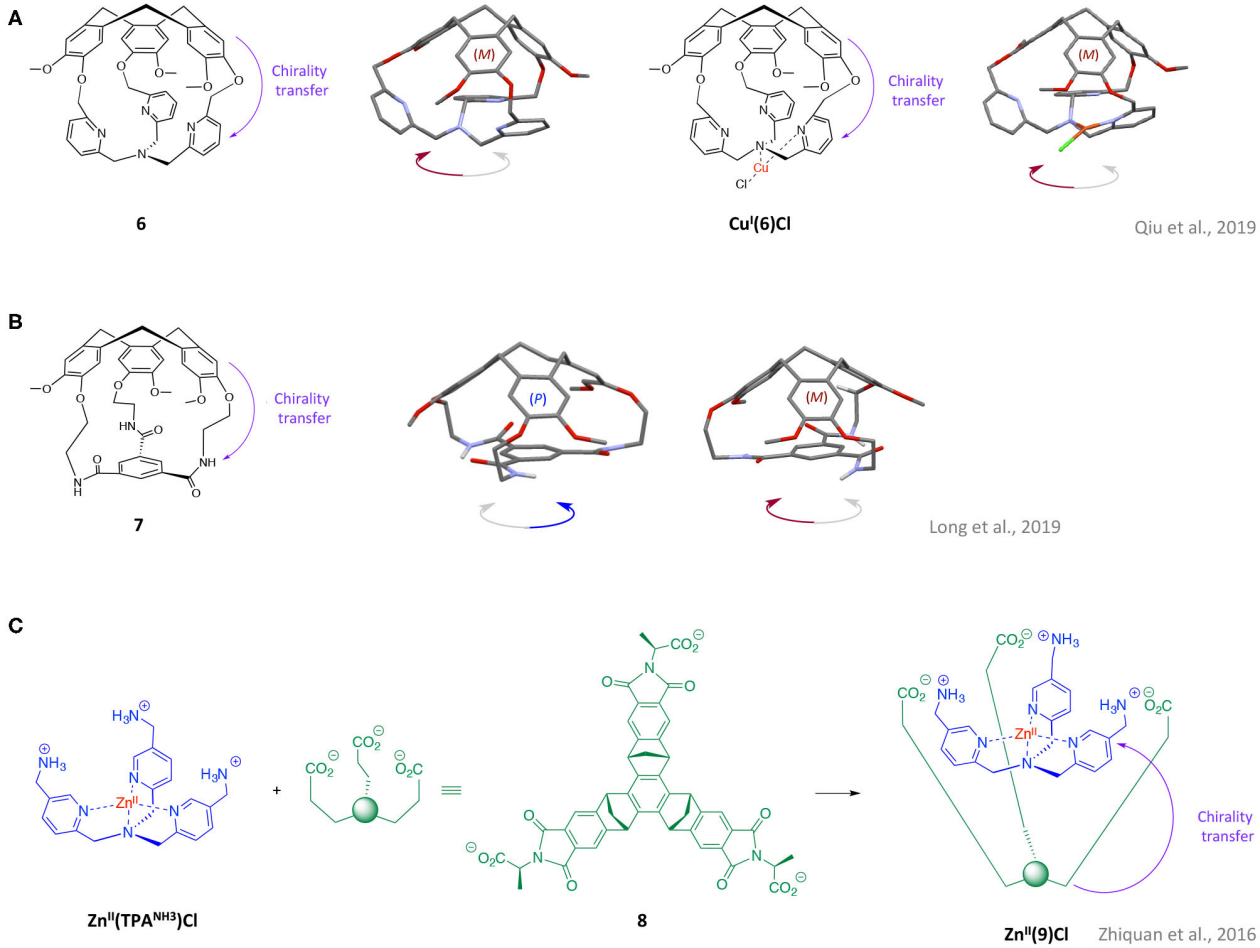
Examples of *C*_3_ symmetrical cages showing a propagation of the stereogenic information from a chiral unit to another tripodal moiety. Schematic representation and XRD structures of hemicryptophane **6** and its corresponding CuCl complex **(A)** and hemicryptophane **7 (B)**, along with the chemical structures of the nesting complex **Zn**^**II**^**(9)(Cl)** and its precursors **8** and **Zn**^**II**^**(TPA**^**NH3**^**)Cl (C)**. Purple arrows depict the observed chirality transfers.

Based on these interesting examples of chirality transfer between chiral unit and linkers, the supramolecular chemists asked themselves: could this phenomenon be extended in order to induce and control the helical arrangement of another *C*_3_ symmetrical unit? The remote control of the helical arrangement of some tripodal units is indeed of particular interest, as it might allow turning achiral artificial ligands into enantiomerically-pure binding sites. For example, the control of the helicity of the *C*_3_ symmetrical tris(2-pyridylmehyl)amine (TPA) ligand as attracted considerable attention due to its versatile applications ranging from bio-inspired models (Borrell et al., 2019), catalysts (Peterson et al., 2011), to chiral sensors (You et al., 2011). This ligand could display a propeller-like arrangement of its pyridine units that rapidly interconvert between clockwise and anticlockwise enantiomeric conformations. Therefore, by controlling the sense of the pyridines twist, the achiral TPA could be switched into highly desirable chiral coordinating structures. In 2019, the X-ray structure of the TPA-based hemicryptophane **5** was reported during the study of this cage-ligand for selective metal-based methane oxidation (Ikbal et al., 2019). This solid-state structure reveals a CTV-dictated triple-stranded helical arrangement of the phenyl linkers of **5**, but no orientation of the southern TPA part was observed (Figure 1E). On this basis, Colomban, Martinez and co-workers have designed the structurally contracted cage **6** (Qiu et al., 2019) where the phenyl linkers are replaced by single methylene –CH_2_- (Figure 2A). The authors resonated that a closer proximity between the chiral northern CTV cap and the southern TPA ligand would result on the propagation of the chirality to the ligand, allowing for a predictable control of its helicity. These expectations turn out to be accurate since, remarkably, the organic cage **6** displays a controlled clockwise/anticlockwise propeller arrangement of the TPA unit, dictated by the chirality of the CTV unit. The covalent capping with a *M*-CTV unit indeed results in a left-handed propeller arrangement of the TPA (while the *P*-CTV leads to a right-handed propeller arrangement). It should be noted that enantiopure versions of (*M)*-**6** and (*P)*-**6** were obtained in a highly efficient purification step based on the chiral-HPLC resolution of the racemic mixture **(±)6** (ee values >99.5%). Importantly, this strong chirality transfer is maintained upon metallation of the TPA moiety with the Cu(I) metal ion. The resulting complex **Cu**^**I**^**(6)(Cl)**, (Figure 2A) displays a rare T-shaped coordination geometry along with a controlled helicity of the TPA unit that both occur in solution and solid state (XRD, ^1^H-NMR, ECD analysis). The design of the cage-ligand **6** therefore allowed the authors to report an unprecedented Cu(I) complex with a controlled helicity of the TPA-Cu(I) core, highlighting the promises of the approach for the preparation of novel optically pure metal-based catalysts and receptors.

This strategy combining a chiral CTV cap and short spacers to control the chirality of another *C*_3_ symmetrical unit, has been further exemplified by Martinez and his team through the preparation of the hemicryptophane **7** (Long et al., 2019). Due to its ability to engage strong hydrogen bonds, the benzene-1,3,5-tricarboxamide (BTA) unit has been widely reported as useful building block for the preparation of supramolecular assemblies (Kulkarni and Palmans, 2017; Zimbron et al., 2017). Therefore, methods able to tune and control its structural properties are of particular interest. With such considerations in mind, **7** was built aiming at controlling the sense of rotation of the three amides of the BTA unit. This cage consists in a southern BTA covalently linked to a chiral CTV cap by three ethylene –C_2_H_4_- linkers (Figure 2B). Interestingly, the close proximity between CTV and BTA unit in **7**, results in a remote control of the Λ/Δ orientation of the three amides of the south part dictated by the chirality of the CTV cap. It was indeed demonstrated, though XRD analysis of the racemic mixture of **(±)7**, that the BTA unit capped with a *M*-CTV displays a Λ orientation of their amides, while its *P* analog displays a Δ arrangement. It was shown that the enantiopure cages (*M*-Λ)-**7** and (*P*-Δ)-**7** could be easily obtained, with excellent *ee* values (*ee* >*97.5 %*) through straightforward chiral-HPLC resolution of the racemic mixture. Finally, the authors proposed, based on ^1^H-NMR observations, that the transfer of chirality observed in the solid states might be retained in solution. Interestingly, this cage **7**, was used as the chain capper of BTA based supramolecular polymers allowing to control their length: whereas the external face of the BTA unit of **7** interacts with the polymer, the CTV unit crowds the other face, preventing further polymerization (ter Huurne et al., 2020).

Another remarkable strategy to generate and control chirality on an achiral tripodal ligand consists in its non-covalent wrapping with a chiral concave open-cage structure, through intermolecular ionic contacts. This so-called “Russian nesting doll” approach have been reported in 2016 by Badjic et al. which used the chiral molecular baskets developed in their team to create and control a propeller-like arrangement of a TPA-based Zinc complex (Zhiquan et al., 2016). The self-assembled architecture **Zn**^**II**^**(9)(Cl)** is based on supramolecular ionic interactions between a zinc complex substituted with three positively charged ammonium groups **Zn**^**II**^**(TPA**^**NH3**^**)Cl**, and the chiral molecular nest **8** (displaying three negatively charged carboxylates at its rim) (Figure 2C). Formation of the entrapped Zn complex **Zn**^**II**^**(9)(Cl)** in its nesting form, was confirmed by ^1^H-NMR titration, ESI-MS analysis, and computational simulations. The preferential formation of the Russian nesting dolls conformation of **Zn**^**II**^**(9)(Cl)** was explained by both hydrophobic effect and non-polar interactions between the two hydrophobic shell of **8** and **Zn**^**II**^**(TPA**^**NH3**^**)**. It was observed that the chirality of the anionic basket **8**, which displays (S)-alanine amino acids groups at its rim, is efficiently transfer to the cationic **Zn**^**II**^**(TPA**^**NH3**^**)Cl** moiety, resulting in a controlled propeller-like arrangement of the TPA's pyridines. Careful analysis of computed structures and the circular dichroism (CD) spectrum, indeed demonstrated that the supramolecular interactions between the three carboxylates of **8** and the ammoniums of **Zn**^**II**^**(TPA**^**NH3**^**)Cl** were responsible for the exclusive formation of a left-handed (*M*) Zn-TPA core. Interestingly, this study allowed the authors to demonstrate that a predictable control of the helical arrangement of TPA-based complexes, could be achieved by an ionic contract-based transfer of chirality. In 2017, the same group further exemplified the approach by studying the capture of several metallated and non-metallated TPA derivatives by both anionic and cationic molecular baskets (Zhiquan et al., 2017).

## Conclusion, Discussion, and Future Directions

To summarize, this mini-review highlights recent advances related to chirality dynamics within tripodal supramolecular cages in terms of induction, transfer and control of the chiral information. Various hosts of *C*_3_ symmetrical type, displaying a predictable and robust control of the chirality, have been recently prepared. The tendency of tripodal structures to form triple-stranded helix or propeller-like arrangements has been exploited to prepare architectures displaying an internal transfer of the stereogenic information from one chiral unit to (i) its nearest linkers or (ii) another *C*_3_ symmetrical unit. This predictable way to generate and control chirality on another linked unit was found to arise from non-covalent interactions of different nature, such as hydrogen-bonding or steric repulsion. Interestingly, this approach has been used to turn achiral ligand into highly valuable coordinating structures, leading to metal complexes with controlled chiral environments. These strategies open new ways in four main research topics. The propagation of the chirality along linkers and even to the opposite face of the cage could lead to highly efficient enantioselective sensors, due to the presence of a strongly controlled chiral environment around the guest-binding site. Indeed, although examples of *C*_3_-symmetrical cages displaying enantioselectivity in the recognition of chiral guests remain rare (Sambasivan et al., 2010; De Rycke et al., 2018), it has been recently shown that remarkable enantioselective recognition of chiral neurotransmitters or carbohydrates can be reached by hemicryptophane cages presenting a *C*_3_ symmetrical axis (Long et al., 2018b; Yang et al., 2020). Secondly, by controlling the chirality at both first and second coordination sphere levels of metal complexes, promising chiral confined catalysts for enantioselective transformations could be obtained. This represents a highly challenging goal since, to the best of our knowledge, there is no example of chiral *C*_3_ symmetrical cages able to induce enantiomeric excess when used as asymmetric catalyst. This approach could also provide new tools for controlling the chirality and the length of helical supramolecular polymers by acting as enantiopure cappers. Finally, the combination of chiral *C*_3_ units with achiral fluorescence units, or lanthanide complexes, could lead to the construction of new fluorescent hosts with a chiral environment around the fluorophore that is fully imposed by the enantiopure tripodal unit (chirality transfer), giving new structures for CPL applications.

Altogether, these examples of preparation of enantiopure chiral *C*_3_ symmetrical supramolecular cages have led to the discovery and understanding of the mechanisms that result in chirality dynamics. It is therefore reasonable to expect that such way of generating, controlling, and propagate chirality will be further applied to other class of supramolecular architectures, and will result in the preparation of new kinds of stereoselective and adaptive hosts. In particular, aiming at mimicking the allosteric properties of biological receptors, the chirality dynamics in well-defined cages is the key to develop challenging on-demand control of the hosts chirality, dictated by the nature of the encapsulated guest (Bravin et al., 2019; Pavlovic et al., 2019).

## Author Contributions

GQ, PN, CC, and AM have co-written the paper. All authors discussed the results and commented on the manuscript.

## Conflict of Interest

The authors declare that the research was conducted in the absence of any commercial or financial relationships that could be construed as a potential conflict of interest.
